# Numerical modeling as a tool for evaluating the renewability of geothermal resources: the case study of the Euganean Geothermal System (NE Italy)

**DOI:** 10.1007/s10653-021-01028-4

**Published:** 2021-07-16

**Authors:** Filippo Torresan, Leonardo Piccinini, Mauro Cacace, Marco Pola, Dario Zampieri, Paolo Fabbri

**Affiliations:** 1 Department of Geosciences, Università degli Studi di Padova, Padova, Italy; 2 Geothermal System Hydrostructures (GSH), Interdepartmental Centre “Giorgio Levi Cases” for Energy Economics and Technology, Università degli Studi di Padova, Padova, Italy; 3 Helmholtz Centre Potsdam – GFZ German Research Centre for Geosciences, Telegrafenberg, Potsdam, Germany; 4 Croatian Geological Survey, Zagreb, Croatia

**Keywords:** Renewability assessment, Geothermal system modeling, Unstructured mesh, Hydrogeological reconstruction, Euganean Geothermal System

## Abstract

Renewable natural resources are strategic for reducing greenhouse gas emissions and the human footprint. The renewability of these resources is a crucial aspect that should be evaluated in utilization of scenario planning. The renewability of geothermal resources is strictly related to the physical and geological processes that favor water circulation and heating. In the Veneto region (NE Italy), thermal waters of the Euganean Geothermal System are the most profitable regional geothermal resource, and its renewability assessment entails the evaluation of fluid and heat recharge, regional and local geological settings, and physical processes controlling system development. This renewability assessment is aimed at defining both the importance of such components and the resource amount that can be exploited without compromising its future preservation. In the second part of the twentieth century, the Euganean thermal resource was threatened by severe overexploitation that caused a sharp decrease in the potentiometric level of the thermal aquifers. Consequently, regulation for their exploitation is required. In this work, the renewability of the Euganean Geothermal System was assessed using the results from numerical simulations of fluid flow and heat transport. The simulations were based on a detailed hydrogeological reconstruction that reproduced major regional geological heterogeneities through a 3D unstructured mesh, while a heterogeneous permeability field was used to reproduce the local fracturing of the thermal aquifers. The model results highlight the role played by the resolved structural elements, in particular the subsurface high-angle faults of the exploitation field, and by the anomalous regional crustal heat flow affecting the central Veneto region.

## Introduction

Geothermal energy is a renewable energy, and the use of geothermal resources for industrial, medical, recreational, heating, and electricity purposes is constantly increasing (Lund & Boyd, 2016). The renewability of these resources, their sustainable utilization, and the control of geological and hydrogeological processes on the development of geothermal systems are widely discussed topics (Axelsson, 2010; Bense et al., 2013; Curewitz & Karson, 1997; Faulds et al., 2013; Mongillo & Axelsson, 2010,2010; Monterrosa & Montalvo López, 2010). According to Stefansson (2000) and Rybach (2007), renewability requires that the amount of thermal energy or fluids removed from the reservoir are continuously replaced. Therefore, renewability is an intrinsic property of the resource that depends on the regional and local geological and hydrogeological settings of the geothermal system associated with the resource and, in particular, on the fluid and heat recharge rates and the hydraulic and thermal properties of the reservoir.

However, anthropogenic utilization (i.e., exploitation) can be greater than the renewability of the system in depleting resources. Therefore, exploitation sustainability is a prominent aspect of geothermal resources in which human action plays a significant role in their long-term preservation (Axelsson, 2010; Axelsson et al., 2004; Rybach, 2007; Rybach & Eugster, 2010; Satman, 2010; Stefansson, 2000). When a geothermal system is overexploited, sustainability can achieved by reinjecting the exploited waters to maintain the production level over a long time (Huo et al., 2019; Kaya et al., 2011; Li et al., 2019; Limberger et al., 2018; Rivera Diaz et al., 2016; Su et al., 2018). However, reinjection is not always energetically or economically effective, and preservation of the resource can only be achieved through proper exploitation management based on the renewable component of the geothermal system (Axelsson et al., 2004; Rybach, 2007; Shortall et al., 2015).

Due to the concomitant interaction of several processes, the assessment of renewability can be very complex. In this regard, numerical simulations are a helpful tool to reproduce the physical, chemical, and mechanical processes affecting a geothermal system and to assess the impact of geological and hydrogeological settings on the magnitude of these processes (Blöcher et al., 2010; Franco & Vaccaro, 2014; Gunnarsson & Aradóttir, 2015; Iorio et al., 2020; Llanos et al., 2015; O’Sullivan et al., 2010; Pandey et al., 2018). The application of numerical modeling for this purpose is widely employed in the literature (e.g., Baiocchi et al., 2013; Borović et al., 2019; Dempsey et al., 2012, 2015; McKenna & Blackwell, 2004; Montanari et al., 2017; O’Sullivan et al., 2001; Porras et al., 2007; Viaroli et al., 2019; Volpi et al., 2017; Wisian & Blackwell, 2004). Considering the geological complexity that is common in geothermal systems, its implementation in numerical simulations is crucial. The discretization of such a complex geological setting is generally a prominent issue that can lead to an inaccurate estimation of the geothermal processes and their impact (Fowler et al., 2016). 3D unstructured meshes have been recently used to represent heterogeneities related to tectonic features or to reproduce geometric details of a reinjection plant (Blöcher et al., 2015; Kim et al., 2010; Salimzadeh et al., 2018; Xing, 2014). Furthermore, site-specific physical property values for populating the model are needed (Borović et al., 2019; Brehme et al., 2016; Guillou-Frottier et al., 2013; Mottaghy et al., 2011). All these aspects and their intrinsic uncertainties (i.e., lateral and vertical variations in the geological setting, partial knowledge of the structures, and incomplete datasets of the properties) have to be considered for a correct and realistic assessment of the renewable component of a geothermal system.

**Fig. 1:**
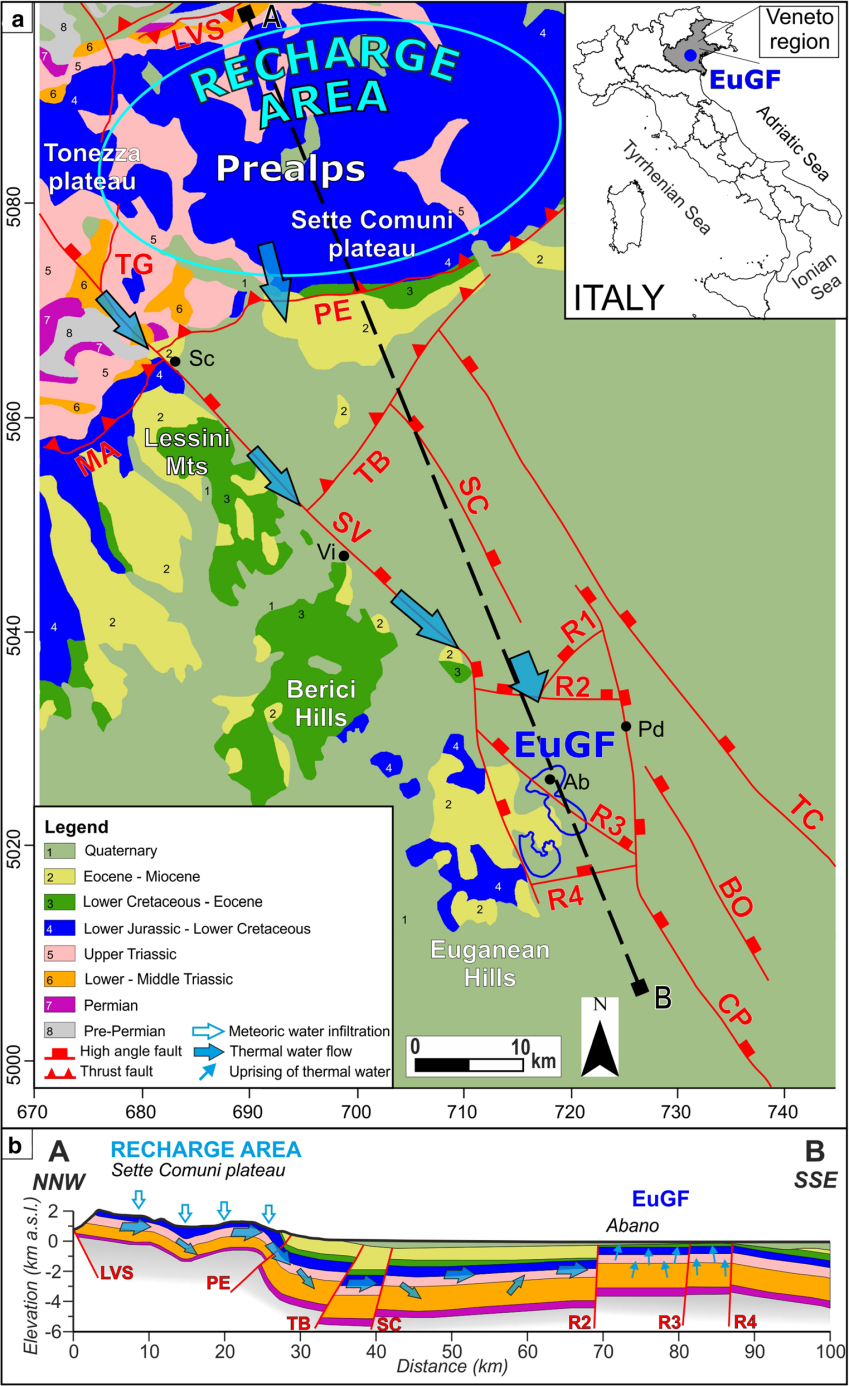
Geological setting of central Veneto and conceptual model of the Euganean Geothermal System. The waters infiltrate in the Prealps (the area bordered by the light blue line), flow toward the south in a Mesozoic carbonate aquifer and reach the exploitation area of the Euganean geothermal field (EuGF; the area bordered by the dark blue line). The main fault systems in the study area are shown as follows: Alpine thrusts (MA = Marana thrust; LVS = Val di Sella thrust; PE = Pedemontana thrust; TB = Thiene-Bassano thrust), Schio-Vicenza fault system (TG = Tormeno-Gamonda fault; SV = Schio-Vicenza fault; SC = Sandrigo-Camisano fault; TC = Travettore-Codevigo fault; BO = Bovolenta fault; CP = Conselve-Pomposa fault), and relay ramp faults (R1 to R4). The relevant cities and municipalities (black circles) are Schio (Sc), Vicenza (Vi), Padova (Pd), and Abano Terme (Ab). The kilometric coordinates of the map are given in the UTM zone 32 N system using the WGS84 datum. This figure is modified after Pola et al. (2015b), Pola et al. (2020), and Torresan et al. (2020)

The Euganean Geothermal System is one of the most important water-dominated, low-enthalpy geothermal systems in Italy and southern Europe. It is a regional-scale fault-controlled hydrothermal system in northeastern Italy (Veneto region). Its recharge area is located approximately 100 km north of the exploitation field (Fig. 1), while the exploitation area consists of a band of 25 km^2^ located southwest of Padua known as the Euganean Geothermal Field (EuGF). Approximately 170 wells are currently active and extract approximately 15 M m^3^ of thermal waters per year with temperatures ranging from 63 to 87 °C (Fabbri, 2001; Fabbri & Trevisani, 2005). Thermal waters are mainly used for therapeutic purposes and feed spas located in the municipalities of Abano Terme, Montegrotto Terme, Galzignano Terme, and Battaglia Terme (Fig. 1). The tourism industry that is related to recreational and therapeutic activities produces an income of approximately 300 M Euro per year (Fabbri et al., 2017). Euganean thermal waters have been used since the Roman epoch for therapeutic purposes (Ghedini, 2011) that take advantage of the natural thermal springs. The forced exploitation of thermal waters with wells started in the twentieth century. Initially, the wells exploited the shallow aquifers that are hosted in the alluvial sediments and hydraulically connected with the deeper rocky reservoir. Subsequently, the increase in the water demand led to the drilling of deeper wells into the underlying fractured reservoir. The result was an overexploitation of the resource that reached its peak in the 1960s and 1970s. During this phase, a sharp decrease in the potentiometric level occurred, and the natural thermal springs dried up. Regulations on the exploitation rates that were applied in the 1980s and 1990s have helped the potentiometric level partially recover, although it was not possible to restore it to the pre-exploitation level (Fabbri et al., 2017; Pola et al., 2015a). The evolution of the anthropogenic impact on the Euganean thermal resource and its former endangerment highlight the need to define a sustainable exploitation plan. This need is strengthened the future utilization scenarios of the Euganean geothermal resource, in which the use of the hottest waters for heating and electricity generation is joined with current recreational and balneotherapical purposes.

A previous study demonstrated that sustainable utilization of this resource cannot be achieved by reinjection due to the absence of a high local heat flux, the high permeability of the bedrock, and the amount of drilled wells, which could result in the probable interaction between the exploitation and reinjection wells (Torresan et al., 2020). Consequently, sustainable utilization can only be reached by controlling exploitation based on the renewability of the system. In this study, coupled fluid flow and heat transport numerical simulations are used to assess EuGS renewability. The numerical simulations are focused not only on quantifying recharge in terms of fluid and heat but are also centered on the role played by geological and structural conditions that favor the development and preservation of the system. This approach, allowing a more effective assessment of the system limits, increases the knowledge of EuGS processes favoring the implementation of a future local numerical model focused on exploitation management and the sustainability assessment. Similar numerical approaches are used to address renewability, its correlation with geological processes, and sustainability of exploitation in other orogenic belts of southern Europe and their forelands (Baietto et al., 2008; Dussel et al., 2016; Magri et al., 2012; Saroli et al., 2019; Sonney & Vuataz, 2009; Viaroli et al., 2019). The workflow adopted for the evaluation of renewability comprises different stages. The main geological and structural features affecting the geothermal system were reproduced in a comprehensive 3D regional hydrogeological model (Torresan et al., 2020). The model was discretized by employing a detailed unstructured 3D mesh that provides a more accurate reproduction of regional and local heterogeneities. The mesh was then used to carry out coupled simulations of fluid flow and heat transport. The model was calibrated by comparing the simulation results with the temperature profiles of the thermal wells and the historical records of discharge rates in the thermal springs. The workflow concluded with a comparison between the calibrated simulation and specific simulations by testing the role of hydrogeological and structural features.

## Geological and hydrogeological setting

The central part of the Veneto region is characterized by a peculiar structural setting that favors the development of the Euganean thermal resource. To the north, the eastern Southern Alps are separated from the Veneto foreland by a system of ENE–WSW-trending, NNW-dipping thrusts (Fig. 1a) that accommodated their uplift (Castellarin & Cantelli, 2000). The foreland is subdivided into two structural domains: (i) the undeformed Lessini–Berici–Euganei (LBE) structural high and (ii) the Veneto plain foredeep (Fig. 1a). The transition between these domains is marked by a system of high-angle, NNW–SSE-trending, NNE-dipping faults known as the Schio-Vicenza Fault System (SVFS; Pola et al., 2014a). The SVFS was active during the Mesozoic extensional phase in response to the thinning of the Adria passive margin, which resulted in the thickening and eastward deepening of Mesozoic formations South of Padua, interactions within the SVFS developed a relay ramp that favored kinematic transfer between the regional faults (Fig. 1a; Fossen and Rotevan, 2016; Zampieri et al., 2009).

During the Neogene shortening related to the indentation of the Adria margin into the European plate (Mantovani et al., 2009), the Veneto plain foredeep was affected by several flexural cycles associated with the build-up of both the Eastern Southern Alps and the Northern Apennines (Brancolini et al., 2019; Fantoni et al., 2002; Zattin et al., 2006). The high-angle faults of the SVFS were reactivated with sinistral strike-slip kinematics (Massironi et al., 2006; Zampieri & Massironi, 2007; Zampieri et al., 2003), enhancing the deformation of the former relay ramp. Localized deformation resulted in the fracturing of the relay ramp by a system of NNE–SSW-, ESE–WNW-, and NW–SE-trending tensional and shear fractures (Pola et al., 2011, 2014b; Zampieri et al., 2010). The occurrence of this fracture network is corroborated by its deforming an isolated travertine mound in the Abano center that was formerly the site of the Montirone thermal springs (Pola et al., 2014b).

The stratigraphic setting of central Veneto (Fig. 1a) can be outlined based on literature data combined with stratigraphic logs from deep wells in the Veneto Plain and thermal wells in the EuGF (reaching 5 and 1 km deep, respectively). At the surface, the stratigraphic sequence is closed by Quaternary alluvial sediments with a thicknesses reaching 200 m in the EuGF. The rocky part of the sequence can be summarized as follows: (i) Eocene to Miocene clastic rocks, marly limestones, and mudstones locally intruded by basalts from the Paleogene Lessini–Berici–Euganei magmatic cycle (Bellieni et al., 2010); (ii) Lower Cretaceous to Miocene wackestones, marly limestones, and mudstones; (iii) Upper Jurassic to Lower Cretaceous wackestones and mudstones; (iv) Lower to Middle Jurassic wackestones-mudstones and packstones–dolostones; (v) Upper Triassic dolostones; (vi) Lower to Middle Triassic marly limestones, sandstones, packstones–grainstones, and wackestones locally intruded by Middle Triassic effusive felsic rocks (De Vecchi & Sedea, 1983); (vii) Lower to Upper Permian sandstones, claystones, dolostones, marls, micritic limestones, and evaporitic rocks; and (viii) pre-Permian crystalline basement mainly composed of phyllites and micaschists (Antonelli et al., 1990, 1993; Cucato et al., 2012). In the Euganean area, the stratigraphic sequence is intruded by upper Eocene—lower Oligocene trachytes and rhyolites with secondary basalts and latites (Bartoli et al., 2015).

In the EuGF, the thermal waters are stored in two principal interconnected aquifers hosted in Upper Triassic–Lower Cretaceous carbonates at depths ranging from 300 to 600 m and from 800 to 1,000 m (Pola et al., 2016). The 300–600 m deep aquifer is the most exploited reservoir, with the waters being principally used for balneological purposes and transmissivity values ranging between 13 and 500 m^2^/day (Fabbri, 1997). The stable isotope composition of the waters indicates a meteoric origin and an infiltration elevation of approximately 1,500 m a.s.l. (Gherardi et al., 2000). Based on the geological setting and the mean elevation of the mountainous areas in central Veneto, a favorable recharge area can be identified in the Tonezza and Sette Comuni Plateaus, located eastward of the SV fault (Fig. 1a; Pola et al., 2015b). This area is characterized by a high level of fracturing and a well-developed karst system affecting the outcropping Mesozoic rocks that allow the infiltration of meteoric waters (Aurighi et al., 2004; Barbieri & Grandesso, 2007). Once infiltrated, the southward migration of the groundwater is guaranteed by the permeability of the rock matrix combined with the high permeability of the SV damage zone. The circulation of this water occurs both in the Mesozoic formations (Fig. 1b) and in the Permian evaporitic rocks, as testified by the Ca/SO_4_ ratio (0.46 ± 0.4), which is comparable with the reference value of gypsum–anhydrite (~ 0.42; Gherardi et al., 2000). The fluids reach a maximum depth of approximately 3 km in the reservoir (Torresan et al. 2000), while the secondary circulation in the underlying Permo–Triassic formations can reach a depth of 4 km. Considering the slightly anomalous crustal heat flow in the Veneto foredeep, these circulation depths are in agreement with the reservoir equilibrium temperature (80–100 °C) inferred by the K/Mg geothermometer and slightly approach the temperatures inferred by isotopic fractionation between CO_2_ and CH_4_ (170–245 °C). Deep fluids then rise to the ground surface through a local network of fractures and faults within the relay ramp (Pola et al., 2015b, 2020). Therefore, we can discern different and concurrent processes that are responsible for the warm-up of the circulating water, listed here in order of importance: (i) convective processes driven by regional- and local-scale faults and fractures and (ii) anomalous crustal heat flows ranging between 70 and 80 mW/m^2^ (Pasquale et al., 2014).

## Materials and methods

### Numerical model

The aim of this work is to evaluate both the EuGS renewability and the role of hydrogeological processes in developing the thermal resource by fluid flow and heat transport numerical simulations. Therefore, as a first step, it is important to reconstruct the main regional and local structural elements and the geometry of the reservoir. This is achieved by developing a detailed hydrogeological reconstruction of the EuGS (Torresan et al., 2020). In the second stage, the hydrogeological model is input into the numerical environment in the form of an unstructured 3D mesh constructed with MeshIt software (Cacace and Blöcher 2015). This strategy enables us to maintain an accurate representation of the main heterogeneities (e.g., faults, fractures, and thickness variations of the reservoir) affecting the geothermal system.

In solving coupled fluid flow and heat transport, we rely on the commercial FEFLOW 7.0 simulator. FEFLOW is a finite-element-based simulator used to solve for flow and transport processes in (un)saturated porous and fractured media with local discontinuities represented by discrete fault zones and local fractures (Diersch, 2014).

The explicit discrete fracture approach is employed to reproduce hydrostratigraphic units and regional and local discontinuities (Bundschuh & Suárez Arriaga, 2010). This approach represents the units as an equivalent porous medium (EPM) and the main faults and fractures as discrete elements (DEs). Within the EPM domain, fluid flow is simulated by applying the groundwater flow equation to a confined aquifer, and Darcy’s law is chosen for the fluid motion in the DEs. Our choice is justified by the presence of a potentiometric level that is consistently above the top of the thermal aquifers, both currently and during the overexploitation phase in the last century. The hydraulic parameters for solving the final set of equations consist of hydraulic conductivity (*K*) and specific storage (Ss) for both the EPM and DEs. In addition, the thickness (*d*) of the DEs is required. The thermal properties for both the EPM and DEs are thermal conductivity (*λ*), volumetric heat capacity (*ρc*), and effective porosity (*ϕ*). Additionally, radiogenic heat (*H*) is defined only for the EPM.

The parameters and boundary conditions applied to the numerical model are tested by a transient simulation, referred to in the next section as the reference simulation (RS). We carried out a calibration of the RS by varying both thermal and hydraulic parameters within specific ranges and comparing the numerical results with the temperature distribution inferred from thermal logs in the EuGF wells and from historical data of the flow rates of the Euganean thermal springs. The simulation time is set to 2.5 Ma to achieve numerical stability of the results.

Starting from the RS, additional simulations are carried out to define the role played by different features, which are thought to affect the EuGS. This is done to further validate the proposed hydrogeological conceptual model and to quantify the renewability of the system. These additional simulations are divided as follows.

1. The simulations testing a different recharge area and a different crustal heat flow comprise the following:
  - simulation with a recharge area located west of the SV fault, and
  - simulation with normal crustal heat flow.
2. The simulations testing the structural control include the following:
  - simulation neglecting the influence of the Schio-Vicenza Fault System (SVFS),
  - simulation neglecting the influence of SVFS and relay ramp faults, and
  - simulation neglecting the influence of all tectonic structures.

### Model domain and geometric discretization

**Fig. 2:**
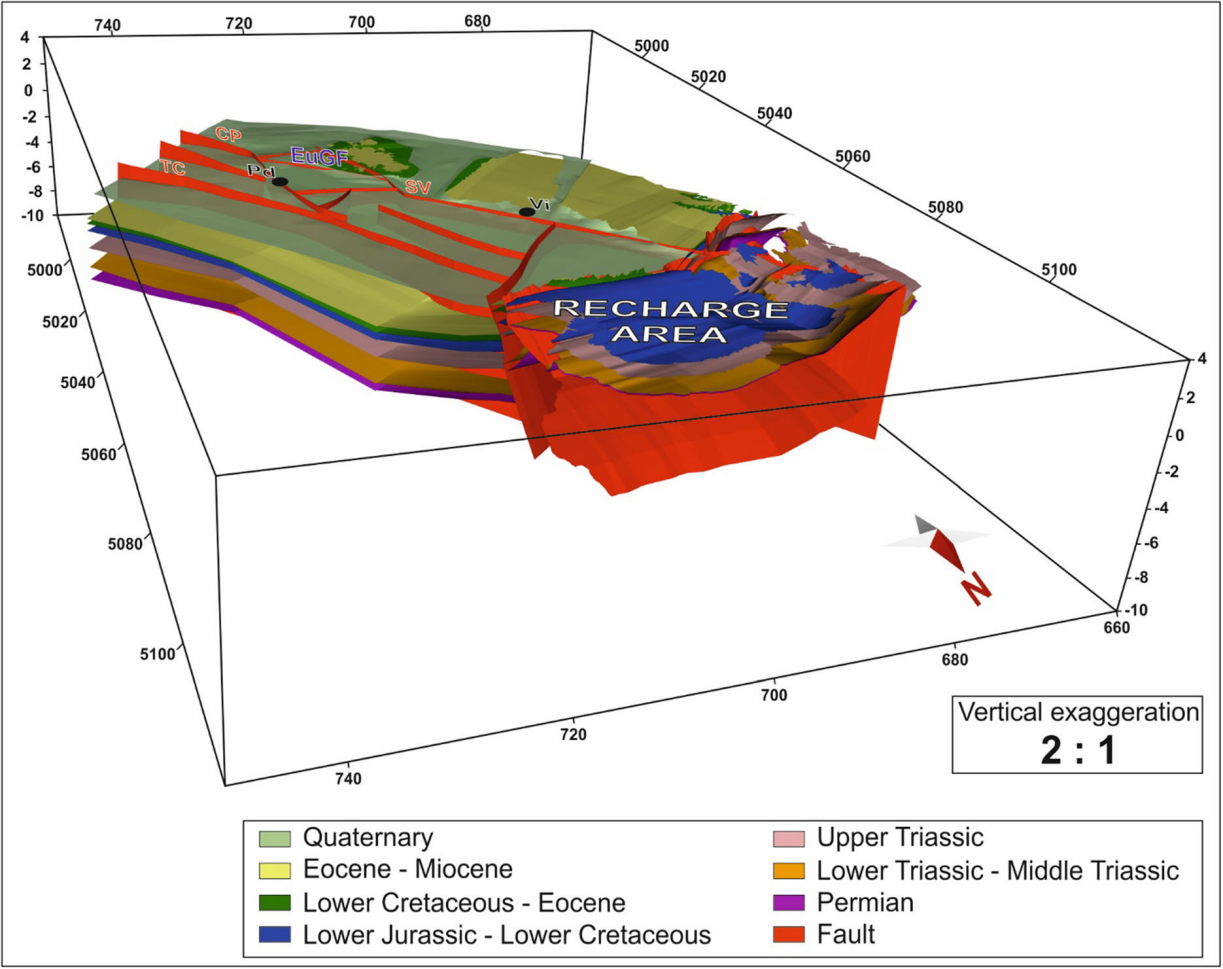
Hydrogeological reconstruction of the central Veneto region (Torresan et al., 2020). The surfaces represent either the bottoms of the hydrostratigraphic units or the regional faults. In particular, the Upper Triassic and the Lower Jurassic-Lower Cretaceous units reproduce the Euganean thermal aquifer. The pre-Permian crystalline basement at the bottom of the sedimentary sequence is not shown. The abbreviations for faults and cities are shown in Fig. 1 as well as the reference to the kilometric coordinate system

As stated by several authors (Pola et al., 2014a, 2020; Zampieri et al., 2009), the geological and structural settings of central Veneto resulting from complex multiphase deformation are fundamental for the existence of the EuGS. Consequently, the geological conditions, which play an important role, demanded accurate implementation in the numerical model. The geological and structural features were reproduced in a 3D hydrogeological reconstruction (Fig. 2; Torresan et al., 2020) made by MOVE software version 7.0 and constrained by a diverse set of data comprising geological and seismic sections, stratigraphic information from boreholes and geological maps (Torresan et al., 2020). In the hydrogeological reconstruction, we included the main features identified as playing fundamental roles in the existence of the EuGS. These can be summarized as (i) the recharge area, (ii) the main regional fault zones driving groundwater flow, and (iii) the exploitation area. In addition, in an attempt to minimize boundary condition effects on the numerical simulations, the hydrogeological model was extended approximately 25 km east and west and 50 km south of the EuGF. Consequently, the size of the model domain was set to approximately 115 km long and 50 km wide. Its thickness was set to 9 km, which included the crystalline basement, because seismic sections analysis located the transition between the sedimentary formations and the basement at a maximum depth of 8 km (Pola et al., 2014a; Torresan et al., 2020).

The SVFS was reproduced as a domain characterized by the presence of high-angle (dip angles ranging between 70° and 87°) NNE-dipping faults according to Pola et al. (2014a), while the faults affecting the interaction zone (R1, R2, R3 and R4 in Fig. 1) were integrated as high-angle, NNW-dipping faults (dip angle of 85°). Finally, the NNW-dipping thrusts located to the north were reconstructed based on available geological sections and structural maps (Antonelli et al., 1990; Pilli et al., 2012).

The geological formations affecting the central part of the Veneto region, from the pre-Permian crystalline basement to the Quaternary sediments, were subdivided into eight hydrostratigraphic units based on their chronostratigraphic sequence and their hydraulic and thermal properties. Consequently, the chrono-hydrostratigraphic sequence, from the bottom to the top of the model, is given in Table 1.

**Table 1:** Chrono-hydrostratigraphic sequence; thicknesses were derived from the stratigraphic logs of deep wells (VIDEPI Project, 2007)

	Lithologies	Age	Thickness (m)	Hydrogeological features
i	Phyllites and micaschists	Pre-Permian	–	Aquiclude
ii	Clastic and evaporitic-carbonate rocks	Permian	50–450	Aquitard
iii	Dolostones and limestones	Lower Triassic–Middle Triassic	300–1500	Aquitard
iv	Dolostones	Upper Triassic	600–800	Aquifer
v	Limestones	Lower Jurassic–Lower Cretaceous	350–700	Aquifer
vi	Marly limestones	Lower Cretaceous–Eocene	100–450	Aquitard
vii	Clastic rocks locally intruded by volcanic bodies	Eocene–Miocene	800–950	Aquitard
viii	Alluvial sediments	Quaternary	40–1100	Aquitard

**Fig. 3:**
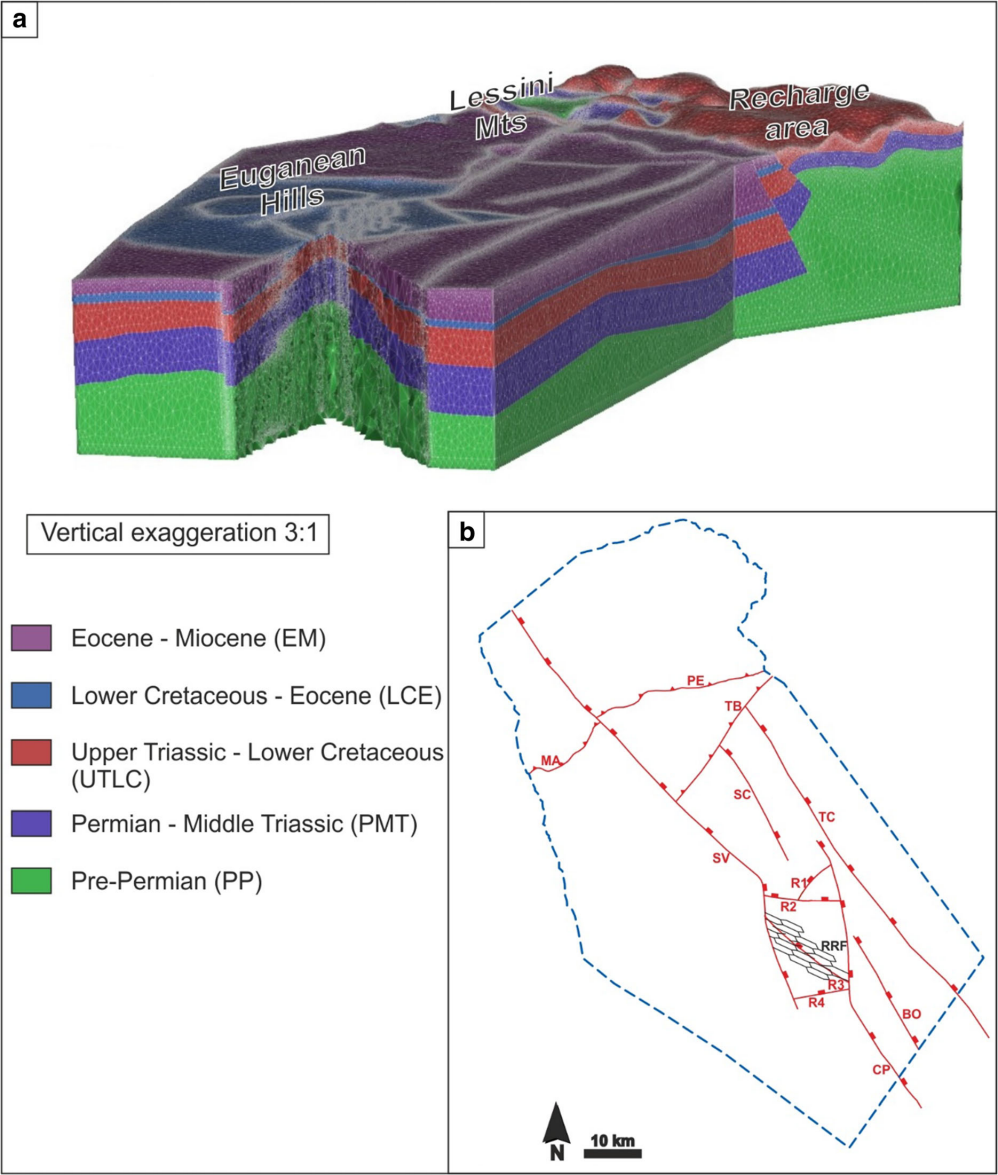
**a** Unstructured mesh used for the hydrogeological reconstruction of the EuGS. **b** Structural sketch showing the regional and local faults (abbreviations given in Fig. 1) and relay ramp fractures (RRFs) implemented in the numerical model. The dashed blue line borders the model domain

In particular, chrono-hydrostratigraphic units (iv) and (v) host the thermal aquifers in the EuGF. The hydrogeological reconstruction (Fig. 2) was discretized into an FEM-consistent model via an unstructured mesh developed by MeshIt software. This approach maintained a high degree of detail in the representation of both the tectonic elements and the unit geometries (Fig. 3a). The input dataset for the meshing was given by (i) the ground surface derived from the digital elevation model (DEM) of the Veneto region (with a 25-m square grid spacing), (ii) the horizons representing the bottom of each hydrostratigraphic unit, and (iii) the 2D planes reproducing the regional faults. These features were implemented as scattered point data in the software, and in a second step, the 3D spatial information for each unit was retrieved by a nonlocal surface reconstruction assisted by an ordinary kriging (OK) method. MeshIt automatically calculated all required intersections and geometric constraints among the different input units, obtaining a volumetric representation of each unit. Based on the meshing strategy, the rock matrix was represented by a 3D unstructured mesh composed of tetrahedral elements that was intersected by faults and fractures that were discretized as 2D triangular boundary conforming surfaces.

During this step, some simplified assumptions were made to reduce the computational efforts during both the meshing and the model processes. Since the groundwater flow in the EuGS occurs mainly in the bedrock, the chrono-hydrostratigraphic unit containing Quaternary alluvial sediments was not considered, and only the rocky formations were modeled. Although there are two exploited reservoirs in the EuGF, they are hydraulically interconnected by the fracture network in the interaction zone. Therefore, we represented them as a single unit by combining the Upper Triassic dolostones and the Lower Jurassic–Lower Cretaceous limestone chrono-hydrostratigraphic units. Similarly, the Permian clastic and evaporitic-carbonate rocks and the Lower Triassic–Middle Triassic dolostone and limestone chrono-hydrostratigraphic units were grouped together. The final result is 5 chrono-hydrostratigraphic units:

i. Pre-Permian chrono-hydrostratigraphic unit (PP);
ii. Permian–Middle Triassic chrono-hydrostratigraphic unit (PMT);
iii. Upper Triassic–Lower Cretaceous chrono-hydrostratigraphic unit (UTLC);
iv. Lower Cretaceous–Eocene chrono-hydrostratigraphic unit (LCE); and
v. Eocene–Miocene chrono-hydrostratigraphic unit (EM).

The UTLC units represent the thermal reservoir.

The damage zone of each fault in the SVFS was reproduced with a thickness of 0.5 km that was calculated from the fault displacement (Pola et al., 2014a, 2020; Savage & Brodsky, 2011). Consequently, the bedrock formations were modeled as an undeformed part (protolith) and a fractured zone (damage zone) surrounding the fault planes. Relay ramp fractures (RRFs) were simulated by a pattern of vertical planes (Fig. 3b) arranged according to the “hill-type” mixed extensional/shear-extensional fracture mesh (Hill, 1977) that deformed both the EuGF subsurface and a travertine mound in the EuGF (Pola et al., 2014b). RRFs extended from the top of the domain to the top of the pre-Permian basement, while regional fault planes extended from the top to the bottom of the domain. The EPM approach was used to simulate the protolith and damage zones, while the planes were implemented as DEs. The mesh was refined in the fractured zones and around the DEs to achieve better characterization of the fluid flow in the parts of the modeling domain that have a prominent role in thermal water circulation. The final 3D unstructured mesh consisted of 895,588 nodes and 5,209,117 tetrahedral elements (Fig. 3a).

### Hydraulic and thermal properties

The hydraulic and thermal properties of the EPM (Table 2) and DE (Table 3) were initially defined based on literature data and analyses carried out in the EuGF or in the central part of the Veneto region and subsequently verified via manual calibration. The hydraulic conductivity of the EPM medium is described as an isotropic tensor, whose *K*_*x*_ and *K*_*y*_ components are reproduced as the horizontal hydraulic conductivity (*K*_*h*_), and *K*_*z*_ is reproduced as the vertical conductivity (*K*_*v*_). The hydraulic conductivity of the thermal aquifers varied between 1.3E-03 and 3.8E-06 m s^−1^, as derived from available pumping tests (Fabbri, 1997). The minimum value, rounded to 4E-06 m s^−1^, was assigned to the *K*_*h*_ of the fractured part of the UTLC unit representing the thermal reservoir. The *K*_*h*_ for the other chrono-hydrostratigraphic units was defined by considering the hydrogeological properties of their lithologies and their roles in the EuGS (Domenico & Schwartz, 1998; Pola et al., 2020). For the LCE and EM units, a value of K_h_ approximately two orders of magnitude lower than the thermal aquifer was assigned because they represent a partial aquitard in the EuGF area. For the PMT unit, a reduction of half an order of magnitude from the thermal aquifer hydraulic conductivity was applied since both chemical analyses and previous numerical modeling point to a secondary circulation in Permian–Triassic units (Gherardi et al., 2000; Pola et al., 2020). The lowest *K*_*h*_ was set to the PP basement unit due to the low permeability of this metamorphic complex. The *K*_*h*_ of the protolith was set two orders of magnitude lower than the fractured part of the chrono-hydrostratigraphic unit. The anisotropy ratio between the *K*_h_ and *K*_*v*_ was set to 100 and 10 for the undeformed and fractured parts, respectively. The hydraulic conductivity in the recharge area was considered isotropic. This assumption simulates the dense fracturing and the well-developed karst system in the recharge area that favors the infiltration of meteoric water. The S_s_ values obtained from available pumping tests ranged from 1.3E-03 to 3.9E-09 m^−1^, and we relied on a value of 1E-05 m^−1^ for the entire model domain.

**Table 2:** Hydraulic and thermal properties of the chrono-hydrostratigraphic units modeled as an EPM

Properties		Eocene–Miocene (EM)	Lower Cretaceous Eocene (LCE)	Upper Triassic–Lower Cretaceous (UTLC)	Permian–Middle Triassic (PMT)	Pre- Permian (PP)
*K*_*h*_ (m s^−1^)	Protolith	1.0E-09	1.0E-09	4.0E-08	8.0E-09	1.0E-10
	Fractured	1.0E-07	1.0E-07	4.0E-06	8.0E-07	1.0E-08
*K*_*v*_ (m s^−1^)	Protolith	1.0E-11	1.0E-11	4.0E-10	8.0E-11	1.0E-12
	Fractured	1.0E-08	1.0E-08	4.0E-07	8.0E-08	1.0E-09
*S*_*s*_ (m^−1^)	–	1.0E-05	1.0E-05	1.0E-05	1.0E-05	1.0E-05
*λ* (J m^−1^ s^−1^ K^−1^)	–	2.2	2.6	3.6	2.4	2.9
*ρc* (MJ m^−3^ K^−1^)	–	2.0	2.0	2.3	2.1	3.0
*H* (W m^−3^)	Protolith	5.0E-07	5.0E-07	5.0E-07	5.0E-07	2.0E-06
	Relay ramp	–	5.0E-07	1.5E-06	5.0E-07	2.0E-06
*ϕ* (%)	Protolith	6.0	7.0	1.0	4.5	1.1
	Fractured	8.5	9.5	3.5	7.0	3.6

*K*_*h*_ horizontal hydraulic conductivity; *K*_*v*_ vertical hydraulic conductivity; *S*_*s*_ specific storage. *λ* thermal conductivity; *ρc* volumetric heat capacity; *H* radiogenic heat; *ϕ* effective porosity

**Table 3:** Hydraulic and thermal properties of the discrete elements (DEs)

	Extensional faults and fractures	Strike-slip fractures
*K* (m s^−1^)	5.0E-03	1.0E-03
*d* (m)	4.0E-02	2.0E-02
*S*_*s*_ (m^−1^)	1.0E-03	1.0E-03
*λ* (J m^−1^ s^−1^ K^−1^)	3.6E + 00	3.6E + 00
*ρc* (MJ m^−3^ K^−1^)	3.0E + 00	3.0E + 00
*ϕ* (%)	100	100

*K* hydraulic conductivity; *d* thickness; *S*_*s*_ specific storage; *λ* thermal conductivity; *ρc* volumetric heat capacity; *ϕ* effective porosity

Regarding the DEs in the relay ramp, their thicknesses were defined based on the measurements taken in the travertine deposit of the EuGF (Pola et al., 2014b). These values were reduced by one order of magnitude from the field data to 0.02 m and 0.04 m for the strike-slip and extensional DEs, respectively, by considering a progressive reduction in the fracture aperture with depth. The regional faults in the SVFS were reproduced with the same thickness as the extensional DEs and by sharing the same kinematics. The maximum value of *K* obtained by pumping tests was assigned to the strike-slip DEs, while the *K* of the extensional DEs was increased by approximately half an order of magnitude because they are more favorable to groundwater flow (Fabbri, 1997; Pola et al., 2014b). Finally, the *S*_*s*_ values for all DEs were fixed equal to the maximum value estimated by pumping tests.

The thermal properties, *λ*, *ρc*, and *ϕ*, of the sedimentary units (i.e., the EM, LCE UTLC, and PMT) were based on their lithologies and using the results of Pasquale et al. (2011). After the initial parameterization, several simulations were performed to constrain the values of these properties. As a result, among the range of values proposed by Pasquale et al. (2011), the minimum values for *λ* and the maximum values for *ρc* were used. The *ϕ* of the protolith was assigned using the minimum values indicated by Pasquale et al. (2011), while it was increased by 2.5% for the fractured bedrock, simulating the greater porosity related to the fault damage zone. For the PP crystalline unit, the thermal properties were assigned with the same approach but based on the results proposed by Vosteen and Schellschmidt (2003).

The radiogenic heat (*H*) was based on the results presented by Pola et al. (2020), where *H* was evaluated by considering the uranium, thorium, and potassium concentrations and the densities of the rocks in Veneto (Faccenda et al., 2007; Germinario et al., 2017; Strati et al., 2015; Tositti et al., 2017). Furthermore, the *H* in the relay ramp was increased to reproduce the higher content of radiogenic isotopes in the Euganean volcanic rocks.

Regarding the DEs, *λ* and *ρc* were assigned by employing the maximum value attributed to the chrono-hydrostratigraphic units. The *ϕ* was assigned a value of 100% considering that the fractures, for the given thickness, are completely open at depth.

### Boundary and initial conditions

**Fig. 4:**
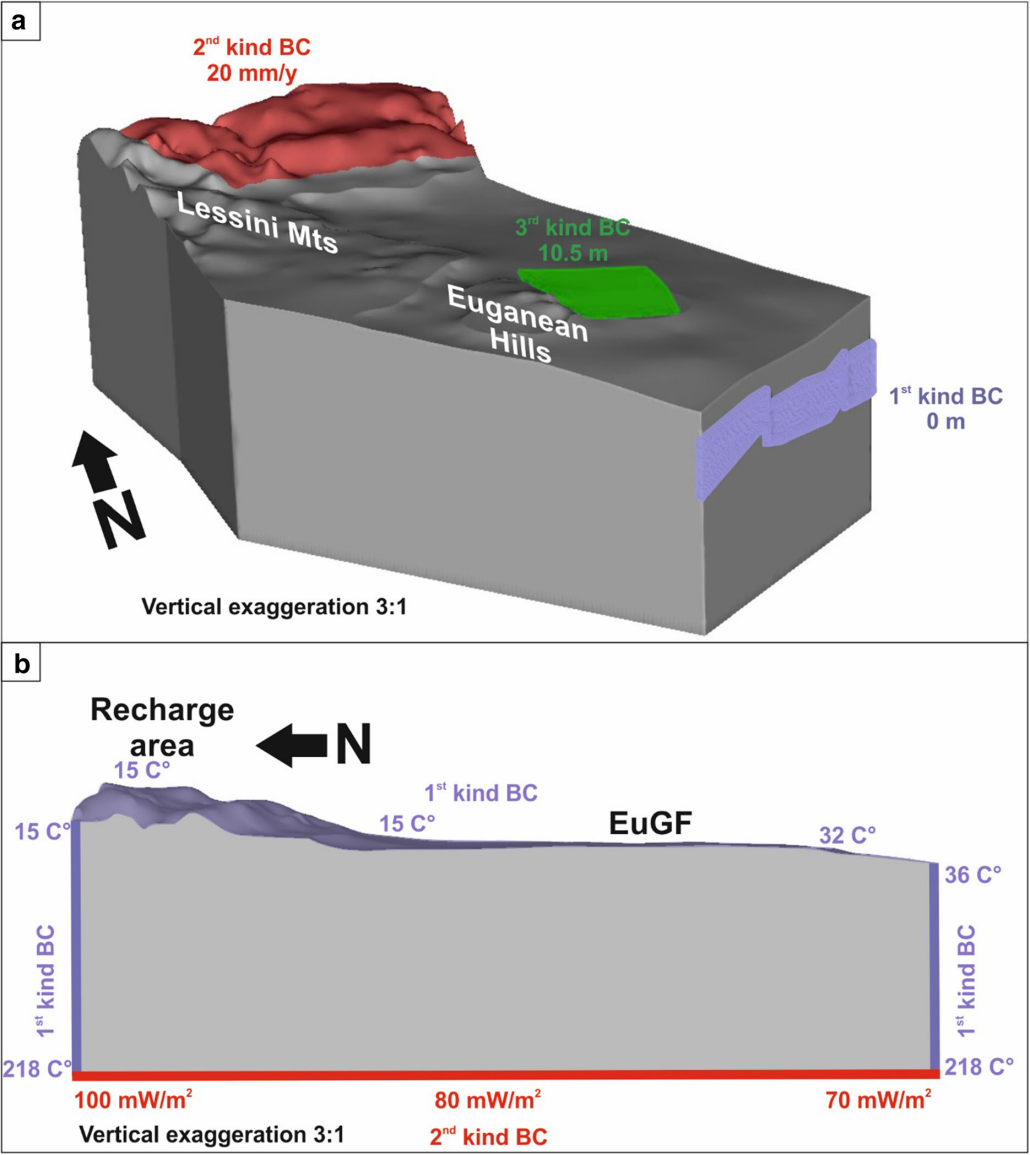
**a** Hydraulic boundary conditions and **b** temperature boundary conditions applied to the model domain

The fluid flow boundary conditions (BCs) applied to the numerical model are as follows (Fig. 4a):

- *Dirichlet-type (1st kind) BC:* this BC is applied in correspondence with the UTLC unit (reservoir) in the southern part of the model domain with a value of 0 m (Fig. 4a). This value corresponds to the mean elevation of the ground level in this part of the domain. It is applied to guarantee the southward migration of groundwater.
- *Neumann-type (2nd kind) BC:* this BC simulates the recharge of the EuGS. It is assigned across the top of the domain in the mountainous area east of the SV fault corresponding to the Tonezza and Sette Comuni Plateaus and surrounding reliefs (Fig. 1). The recharge value is set to 20 mm/y (Fig. 4a). Considering a recharge area of approximately 790 km^2^, this infiltration value corresponds to an inflow of approximately 16 M m^3^/year, which is comparable with the volume exploited currently in the EuGF. The assigned value is lower than the imbalance value of 260 mm/y in the hydrological balance of the Sette Comuni Plateau (Table 4; Aurighi et al., 2004). This choice is related to the fact that the mass balance only considers the outflow from the main system of karst springs at the base of the relief. However, the infiltration also feeds several secondary springs and the alluvial aquifers of the central Veneto Plain, as well as the EuGS.
- *Cauchy-type (3rd kind) BC:* this BC is applied across the top of the domain in the interaction zone with a value of 10.5 m (Fig. 4a), which is the mean elevation of the ground surface in the EuGF area. It is applied to reproduce the outflow of thermal waters in the interaction zone and Euganean thermal springs. A constraint condition allowing only the outflow is added to this BC. The constraint requires an out-transfer coefficient that was set equal to 1.0E-05 s^−1^ for the EPM and to the ratio between the hydraulic conductivity and thickness for the DEs.

**Table 4:** Hydrological balance from Aurighi et al. (2004); *R* and EVT are the mean precipitation and mean evapotranspiration, respectively, calculated over an area of 500 km^2^ during 1961–1990; ER is the effective rainfall calculated as the difference between the *R* and EVT; spring discharge is the average flow of the five major springs emerging at the foot of the Sette Comuni and Tonezza Plateaus; imbalance is the difference between the ER and spring discharge

*R* (mm)	EVT (mm)	ER (mm)	Spring discharge (mm)	Imbalance (mm)
1600	480	1120	820	260

The thermal BCs applied to the model are as follows (Fig. 4b):

- *Dirichlet-type (1st kind) BC*: this BC is applied to the northern and southern borders and to the top of the domain with the exception of the EuGF area and the RRF DEs in the interaction zone (Figs. 3b and 4b). The values are defined using the temperature gradient defined by Pasquale et al. (2014) for the undeformed Po Foredeep and are set equal to 22.6 mK/m. The resulting values are between 218 and 15 °C at the NW limit, between 218 and 36 °C at the SE limit, and between 15 and 32 °C at the top of the domain. The zones excluded from the application of this BC were chosen considering two main factors: (i) the presence of natural hot springs and (ii) the rising hot water from the fractured reservoirs to the Quaternary alluvial aquifer.
- *Neumann*-*type (2nd kind) BC:* this BC is applied at the bottom of the domain and represents the regional crustal heat flow (Fig. 4b). The assigned values range from 100 to 70 mW/m^2^ according to the available heat flow maps (Della Vedova et al., 2001; Pasquale et al., 2014).

The initial conditions, in terms of hydraulic head and temperature distribution, were defined with a preliminary steady-state simulation.

## Results

### Reference simulation

**Fig. 5:**
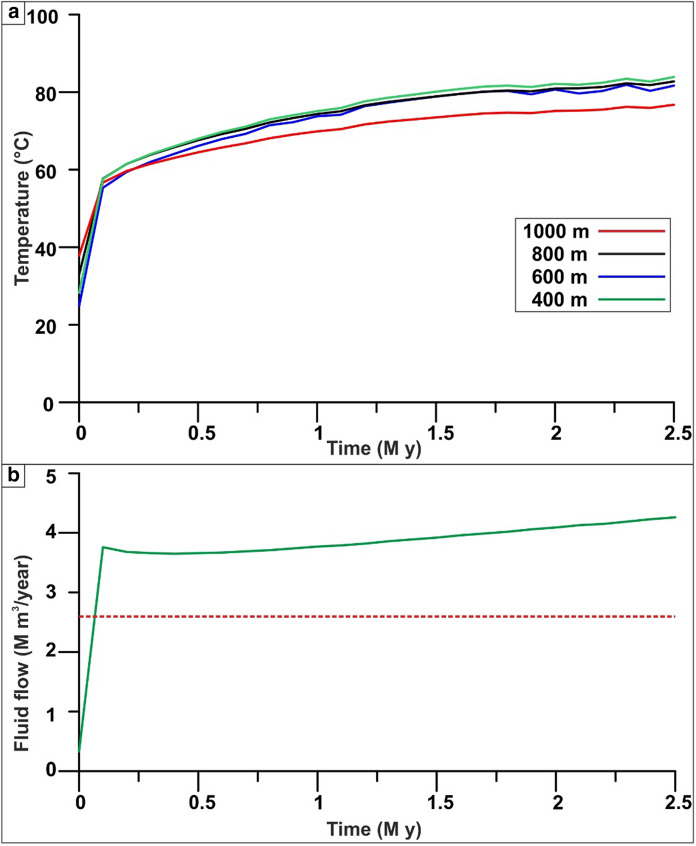
**a** Temperature versus time in the control points located in the thermal reservoir of the EuGF at different depths. **b** Fluid flow through the Cauchy-type boundary condition applied in the interaction zone. The dashed red line represents the threshold value of the volume discharge by thermal springs in the EuGF (2.6 M m^3^/y)

The numerical model was run for a simulation time of 2.5 Ma, reaching a quasi-steady-state condition after 1.5 Ma in terms of the temperature distribution in the EuGF (Fig. 5a), while the numerical stability in terms of fluid flow was reached almost instantaneously (Fig. 5b). The simulation was interrupted at 2.5 M years because the curves “temperature versus time” (Fig. 5a) at different depths within the interaction zone show acceptable stability, and, above all, they achieve a final temperature in agreement with the field data. Anyway, it can be noticed that the increment is drastic during the first time step due to the expected impact of the initial and boundary conditions. The variations decrease progressively over time and after 1.2–1.3 M years are generally lower than 1%. Therefore, the doubling of this simulation time was considered acceptable for achieving a quasi-stationary solution. During the time interval 1.3–2.5 M years, variations higher than the reference value of 1% were observed only: (i) at the depth of 400 m, where the temperature distribution is affected by the boundary effects of the third-type boundary conditions and (ii) at the time step of 2.5 M years, when higher variations could be expected due to the simulation end. These discrepancies can be reasonably accepted, especially considering that the work aims to verify the role played by different factors in the development of the system and considering that this objective is achieved by comparing the temperature distribution of a reference simulation with those derived from the different “geological/hydrogeological” scenarios.

**Fig. 6:**
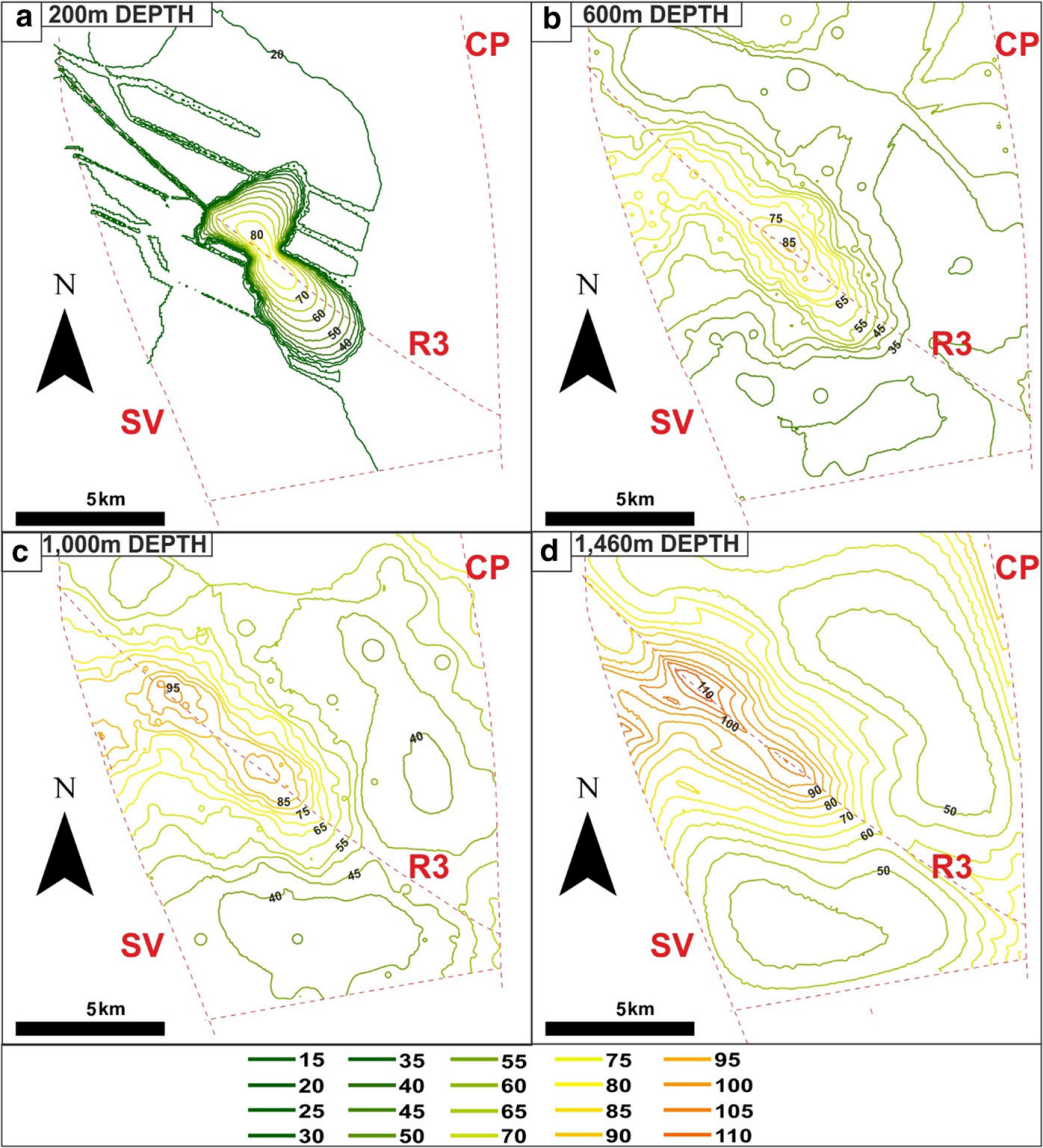
Reference simulation (RS) maps of the temperature distribution in the relay ramp, **a** Temperature distribution at the top of the bedrock formations (200 m in depth). **b** Temperature distribution at the maximum depth of the most exploited reservoir (600 m in depth). **c** Temperature distribution at the maximum depth reached by thermal wells (1,000 m in depth). **d** Temperature distribution at the bottom of the thermal reservoir (1,460 m in depth). A depth interval of 50 m across the reference depths was chosen due to the irregular shape of the unstructured mesh

The results of the reference simulation (RS) were investigated through temperature maps at different depths within the interaction zones (Fig. 6). The maps show the following:

- at the top of the bedrock (200 m deep, Fig. 6a), an increase in the computed temperature corresponds to the EuGF, with values ranging from 25 °C to approximately 80 °C and values less than 35 °C along the tensional fractures and the R3 fault of the relay ramp (see Fig. 3b for the location of faults and fractures).
- At the maximum depth of the most exploited reservoir (600 m deep; Fig. 6b), the temperature distribution shows a NW–SE ellipsoidal shape elongated along the R3 fault trend with maximum values (86.9 °C; Table 5) in the central part of the EuGF.
- At the maximum depth reached by the thermal wells (approximately 1,000 m; Fig. 6c), the shape of the thermal anomaly is comparable with the shallower anomaly, but the maximum temperature value (96.5 °C; Table 5) is recorded in the NW part of the EuGF near the Euganean hills.
- At the bottom of the thermal reservoir (i.e., the bottom of the UTLC unit; 1,460 m deep in Fig. 6d), the temperature distribution is more homogeneous with preferential development along the R3 fault. The maximum temperature at this depth is approximately 110 °C in the NW part of the EuGF.

**Table 5:** Minimum (*T*min), mean (*T*mean) and maximum (*T*_max_) temperatures simulated at different depths in the EuGF

Depth (m)	*T*min (°C)	*T*mean (°C)	*T*max (°C)	*T* measured (°C)
200	19.3	37.5	82.1	38.5–86.1
600	29.2	54.4	86.9	78.9–82.6
1,000	37.0	65.1	96.5	~ 92
1,460	46.3	72.8	107.0	No data

*T* measured represents the range of temperatures inferred by the thermal logs. The investigated depths are described in Fig. 6

**Fig. 7:**
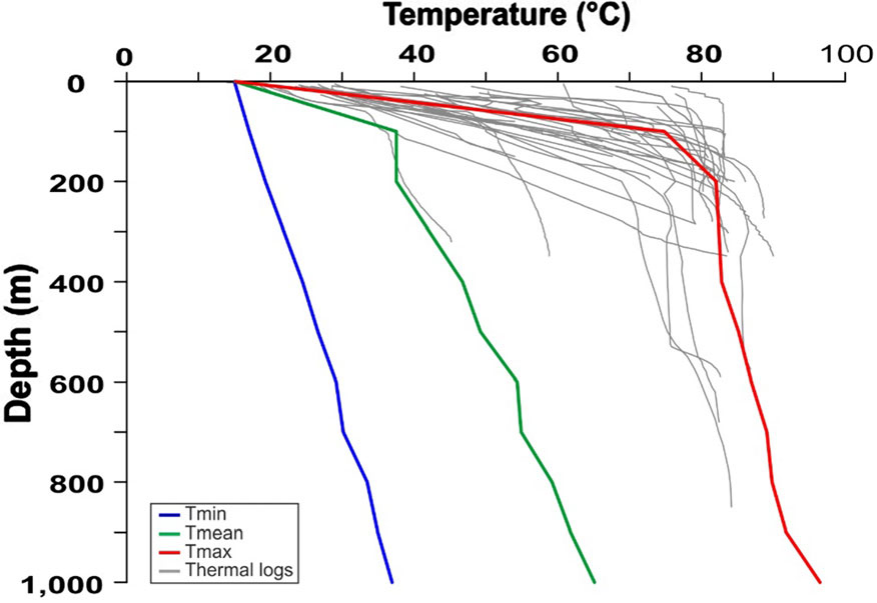
Comparison between the minimum (*T*min), mean (*T*mean) and maximum (*T*max) simulated temperatures and experimental results of the thermal logs

The comparison between observed and simulated temperatures at different depths was carried out for the northern part of the EuGF, including the municipalities of Abano Terme and Montegrotto Terme (Fig. 1). The minimum, mean and maximum simulated temperatures were compared with the temperatures inferred from thermal logs. The results are shown in Fig. 7 and summarized in Table 5 for the most representative depths of the EuGF (Fig. 6). The maximum temperature simulated at a depth of 200 m is comparable to the temperature distributions measured by thermal logs, while Tmean is lower than the minimum value. At a depth of 600 m, the modeled Tmax is slightly higher than the measured temperatures. At a depth of 1,000 m, where a temperature of approximately 92 °C was inferred by the results of a thermal log in an artesian well, the higher modeled temperature matches the experimental data. From a depth of 200 m to the bottom of the thermal aquifer, the temperature gradually increases with a gradient of approximately 0.02 °C/m.

With the same approach, the minimum, mean, and maximum temperatures simulated from the top of the domain to a depth of 1,000 m in the EuGF were graphically compared with thermal logs carried out in the exploitation field (Fig. 7). First, it is evident that Tmin does not match the experimental temperature distribution. This is mainly related to the fact that the minimum modeled values are located near the border of the exploited area that corresponds to the colder portion of the EuGF explored by a few shallow wells. The simulated Tmax reflects the higher values of temperatures measured in the EuGF, with values ranging from 74.8 to 96.5 °C between 100 and 1,000 m in depth, respectively. The simulated *T*mean distribution is comparable to the lower limit of the field data range.

The mean outflow value from the Cauchy-type BC is approximately 3.9 M m^3^/y, with values varying between 3.7 and 4.3 M m^3^/y (Fig. 5b). These values are higher than the total outflow from the thermal springs before the overexploitation of the resource (2.6 M m^3^/y; Mameli & Carretta, 1954). Such a difference can be considered acceptable since the simulated outflow includes the outflow from the bedrock over the entire interaction zone. On the other hand, historical measurements include the flow rates of the main Euganean thermal springs and do not include the inflow of thermal waters from the bedrock into Quaternary alluvial aquifers, as observed in the EuGF and its surroundings.

### Simulations testing the recharge area and crustal heat flow

The impact of the heat flow and the recharge area positioning was evaluated by two additional simulations. These simulations aimed to test the influence of such model components on the development of groundwater flow and temperature distribution within the interaction zone and the EuGF. In these simulations, the hydraulic and thermal properties applied to the EPM and DEs, as well as the initial conditions, were maintained the same as in the RS.

#### Simulation with the recharge area located west of the SV fault

The stable isotope content of the Euganean waters points to an infiltration altitude of approximately 1,500 m a.s.l. that is compatible with the altitudes in most of the Veneto Prealps. In this simulation, the recharge area was maintained in the Prealps, but it moved from east of the SV fault to the west in the Piccole Dolomiti Mountains (Fig. 1). This scenario reproduced the first hydrogeological conceptual model of the EuGS proposed by Piccoli et al. (1973). The total inflow through the recharge area was the same as that of the RS, i.e., approximately 16 M m^3^/y.

The results obtained from this simulation provided an unrealistic hydraulic head distribution and an outflow from the 3rd kind BC that was lower than the historical outflow value from the thermal springs. This result is related to the presence of the Marana thrust (Fig. 1) to the west of the SV fault that juxtaposes impervious crystalline rocks in the hanging wall (PP unit) against the carbonate formations of the reservoir in the footwall (UTLC unit), inhibiting groundwater flow. Consequently, the outflow from the Cauchy-type BC and the temperature distribution in the EuGF area do not conform with the field data, thereby providing additional validation to the revised hydrogeological conceptual model of the EuGS (Pola et al., 2013, 2015b).

#### Simulation with normal crustal heat flow

In this simulation, the importance of the anomalous regional crustal heat flow was tested by setting a constant value comparable to the average geothermal flow in continental areas (Pasquale et al., 2014). Consequently, the Neumann-type BC at the bottom of the model domain was modified using a constant value of 60 mW/m^2^.

**Fig. 8:**
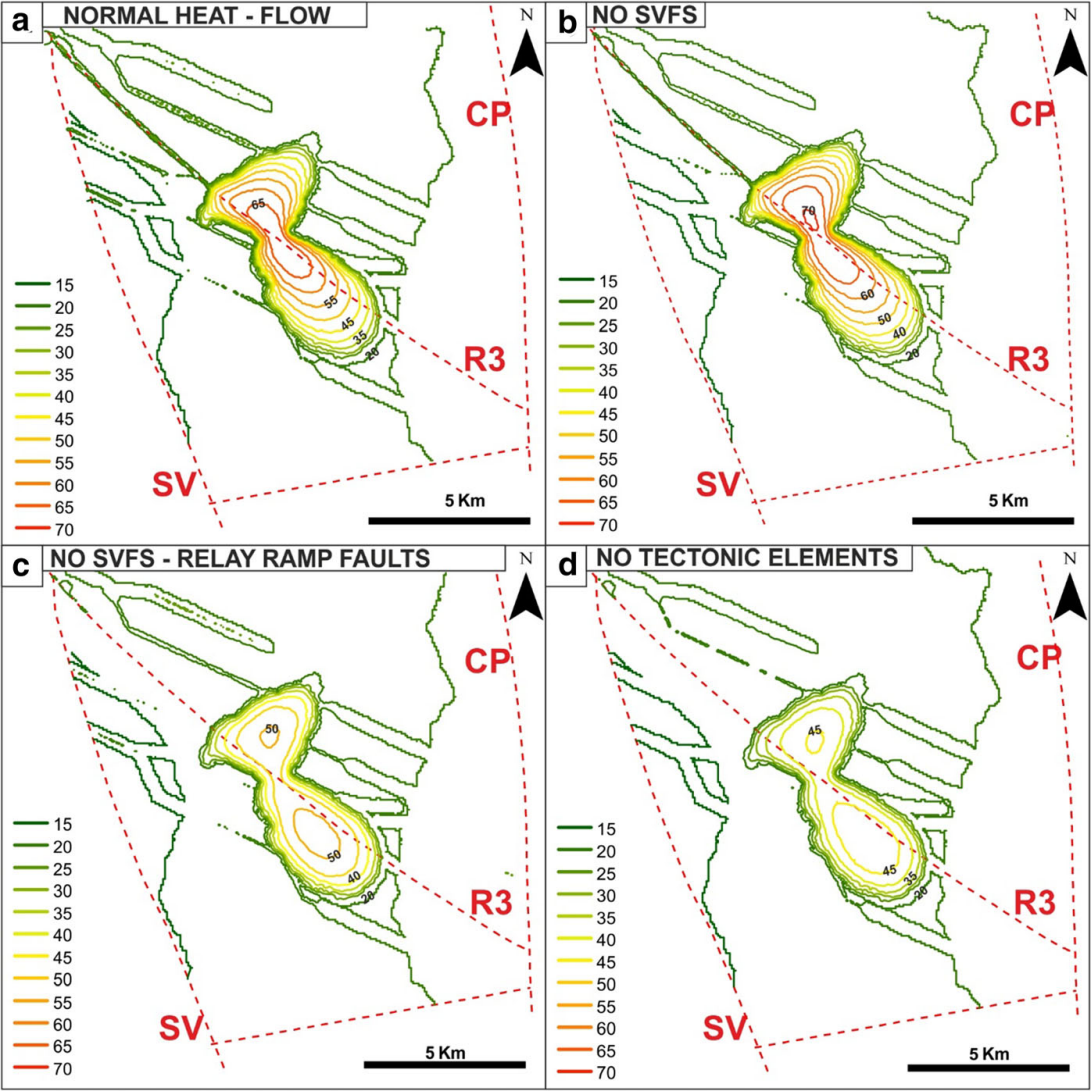
Maps of the temperature distribution in the relay ramp at the top of the bedrock formation (depth of 200 m) for the additional simulations

**Fig. 9:**
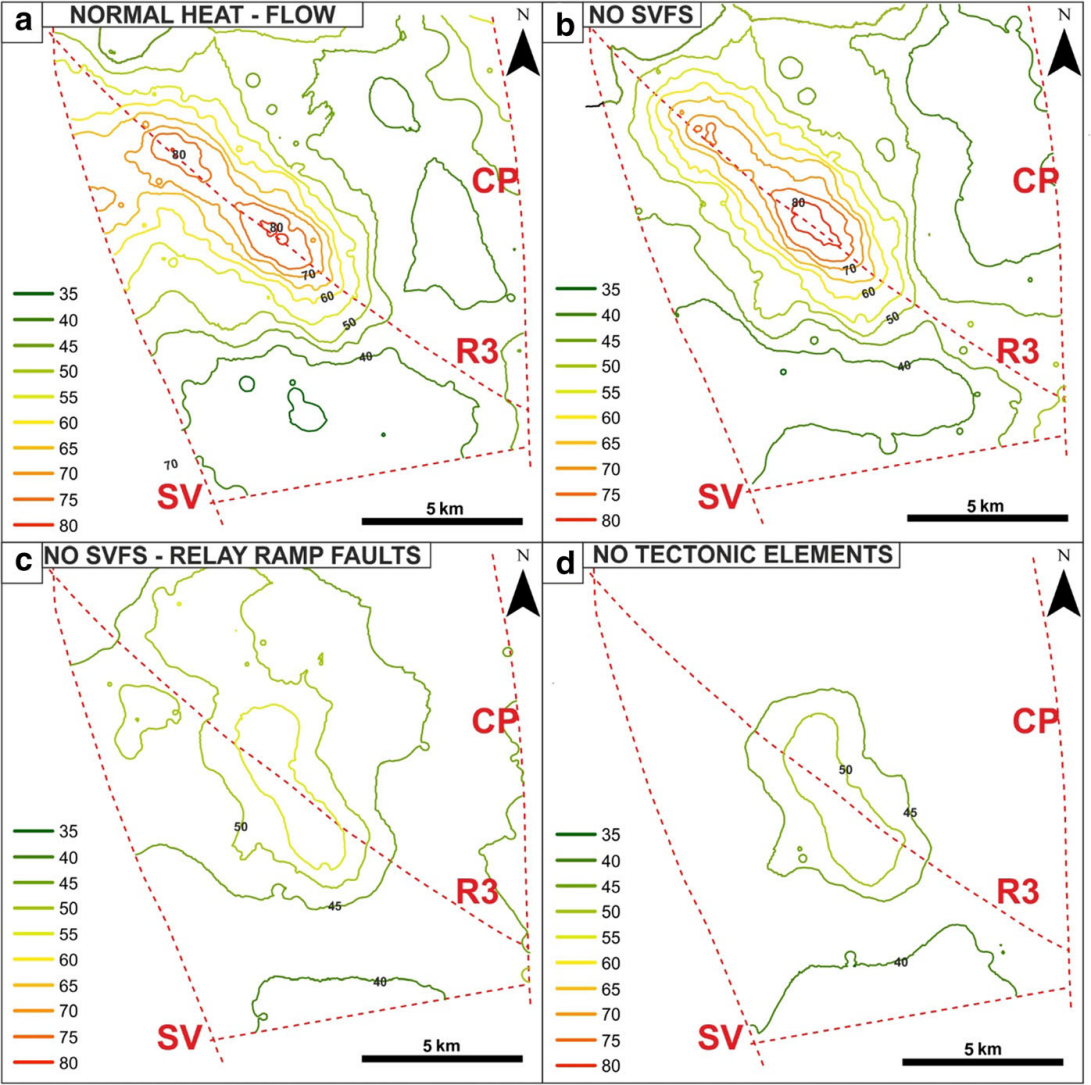
Maps of the temperature distribution in the relay ramp at the maximum depth (1,000 m) reached by thermal wells for the additional simulations

At the top of the domain (Fig. 8a), the temperature ranges from 25 to 65 °C in the EuGF and lower than 30 °C along the fractures within the interaction zone. At 1,000 m in depth (Fig. 9a), the simulated maximum temperature is approximately 80 °C. The thermal anomaly is elongated along the R3 fault with the formation of two areas of positive thermal anomalies corresponding to the EuGF and an area NW of the Abano Terme.

**Fig. 10:**
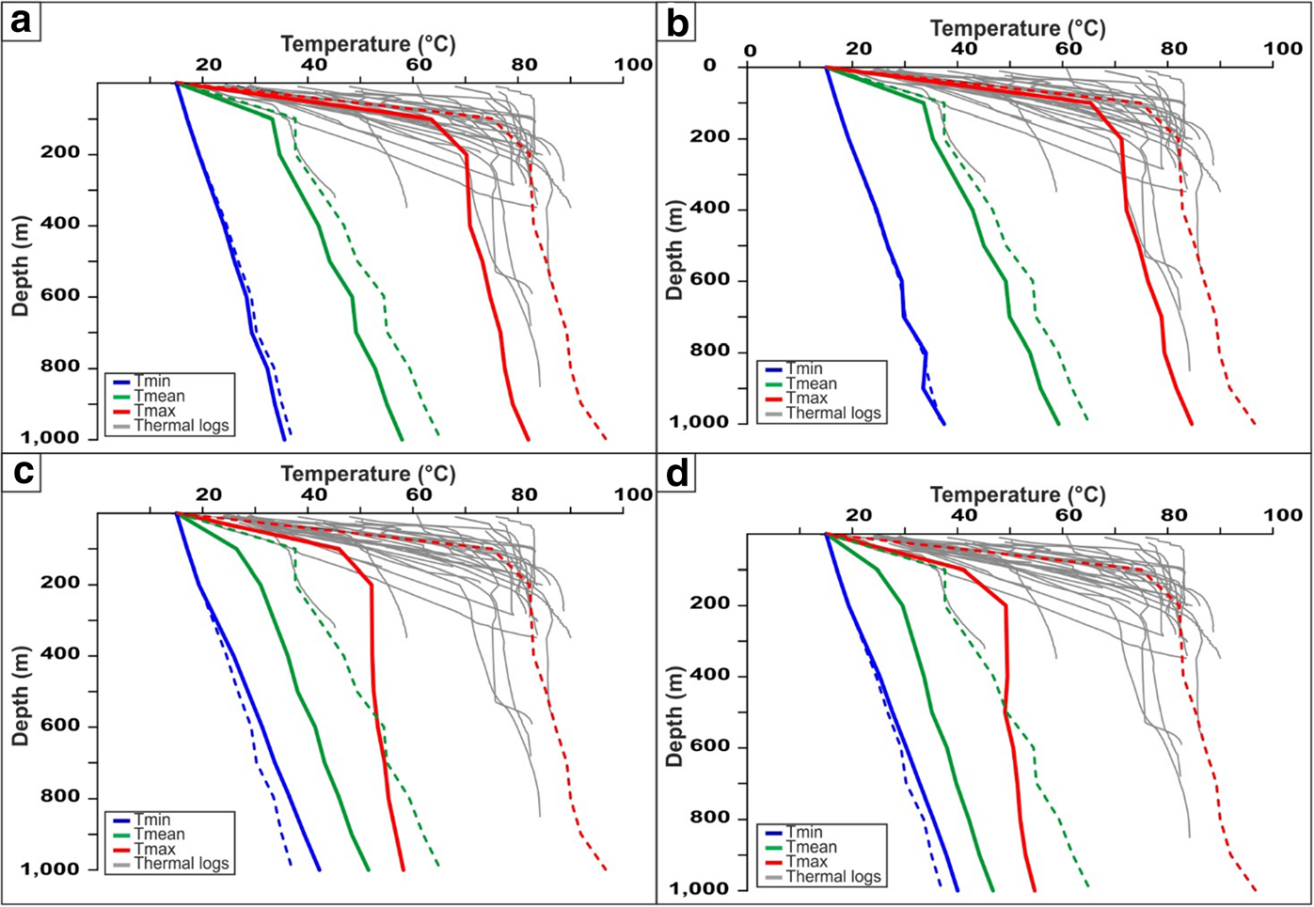
Comparison between the minimum (*T*min), mean (*T*mean), and maximum (*T*max) simulated temperatures and the measurements by the thermal logs. **a** Simulation with normal crustal heat flow. **b** Simulation neglecting the influence of the SVFS. **c** Simulation neglecting the influence of the SVFS and relay ramp faults. **d** Simulation neglecting the influence of all tectonic elements. The dashed lines represent the temperature distribution obtained by the reference simulation (RS)

The modeled values are generally lower than both the RS results and the field data (Table 6 and Fig. 10a), and only the simulated values at 200 m depth approach the measurements. In particular, Tmax shows the highest reduction in the RS results (Fig. 10a).

**Table 6:**
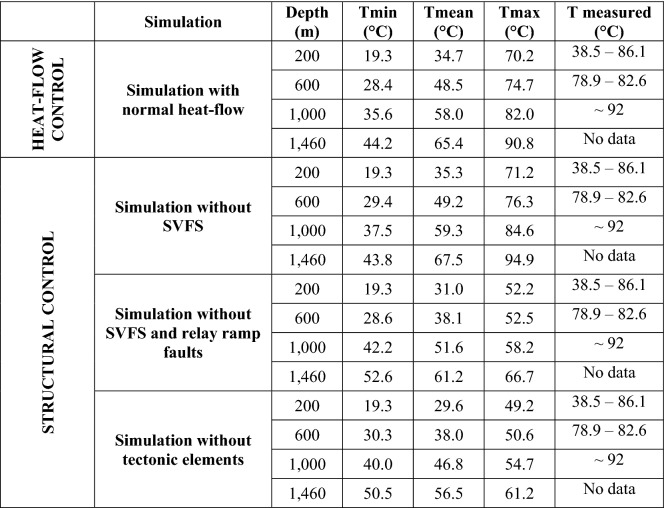
Minimum (*T*min), mean (*T*mean) and maximum (*T*max) temperatures simulated at different depths in the EuGF for the interpretive simulations

*T* measured represents the range of temperatures inferred by the thermal logs at different depths. The investigated depths are described in Fig. 6

**Fig. 11:**
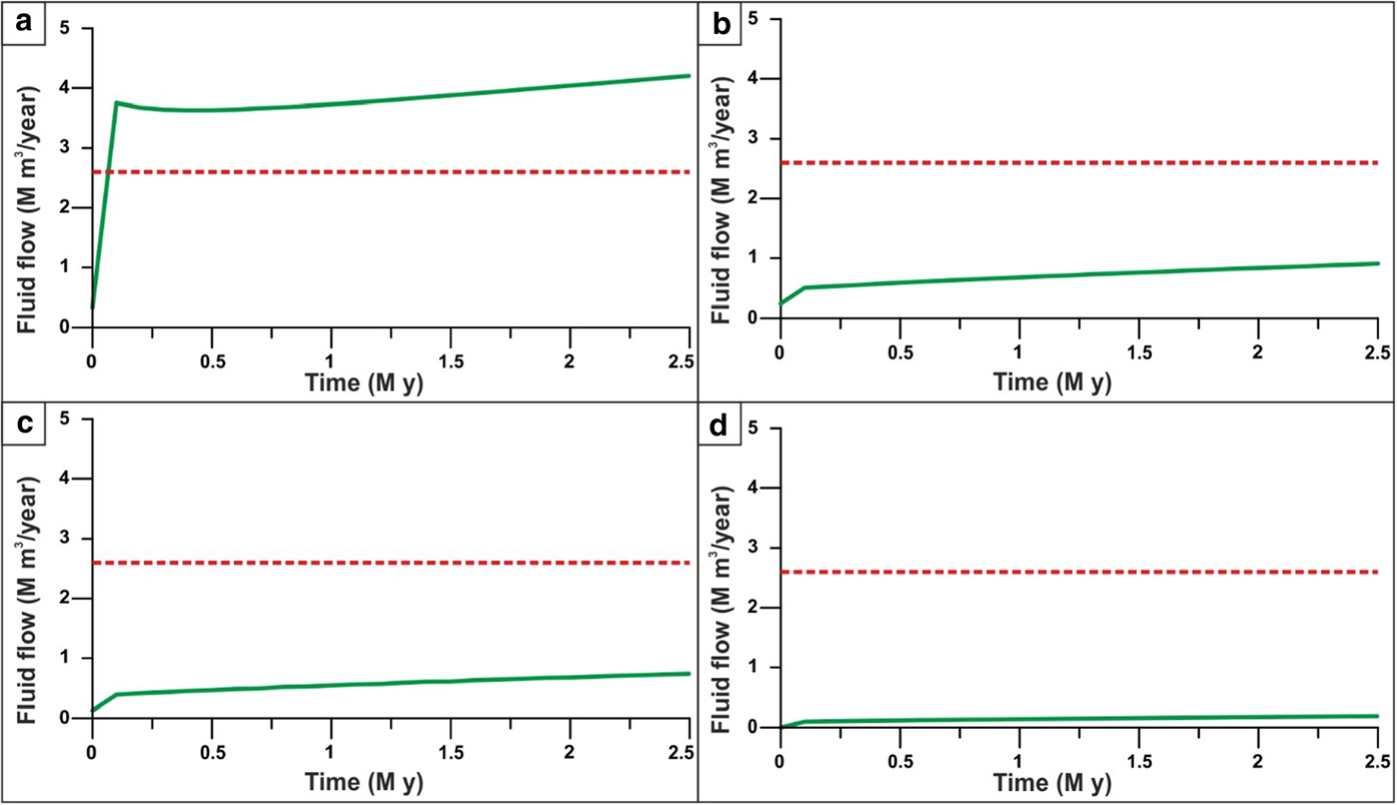
Flow rate through the Cauchy-type boundary condition in the interaction zone. The dashed red line represents the threshold value of the discharge of the thermal springs in the EuGF (2.6 M m^3^/y). **a** Simulation with normal crustal heat flow. **b** Simulation neglecting the influence of the SVFS. **c** Simulation neglecting the influence of the SVFS and relay ramp faults. **d** Simulation neglecting the influence of all tectonic elements

Considering the groundwater outflow from the Cauchy-type BC in the interaction zone (Fig. 11a), we model an outflow rate ranging from 3.6 to 4.2 M m^3^/y, similar to the outflow rate from the thermal springs in the EuGF (2.6 M m^3^/y).

### Simulation testing the structural control

The role played by tectonic structures on the development and renewability of the EuGS was evaluated in three numerical simulations representing different structural settings. All hydraulic and thermal properties and initial and boundary conditions were maintained as in the RS. This assumption is necessary to test the influence of faults and fractures on the groundwater flow and the resulting temperature distribution.

#### Simulation neglecting the influence of the Schio-Vicenza Fault System (SVFS)

The first scenario neglects the incorporation of discrete elements (DEs) for the SVFS while maintaining the high-angle faults and the mesh of the fractures deforming the interaction zone (R1–R4 and RRFs, respectively, in Fig. 3b).

The surface temperature development with values ranging from 25 to 70 °C is limited to the EuGF, while along the fractures, temperatures are lower than 25 °C (Fig. 8b). Moving to the maximum depth reached by thermal wells (1,000 m in depth), the temperature distribution portrays an elliptical shape elongated along the R3 fault (Fig. 9b) with a maximum value of 84.6 °C (Table 6) simulated in the central part of the EuGF.

At 200 m depth, the modeled values are slightly lower than temperatures recorded by thermal logs, while at 600 m and 1,000 m deep, they are systematically lower. These results highlight a reduction in temperature compared to the RS, which is equivalent to the result achieved by the simulation testing the influence of the basal heat flow.

In terms of fluid flow, the absence of the SVFS causes an abrupt reduction in the outflow from the interaction zone, with values ranging from 0.5 to 0.9 M m^3^/y (Fig. 11b). Based on these observations, we can conclude that it is not possible to explain the existence of natural thermal springs located in the EuGF only by considering the control of the faults and fractures affecting the interaction zone.

#### Simulation neglecting the influence of the SVFS and relay ramp faults

In this simulation, we neglect both the influence of the SVFS and the high-angle faults characterizing the relay ramp (R1–R4; Figs. 1 and 3b). Consequently, only the RRF DEs of the interaction zone (Fig. 3b) and increasing bedrock permeability were maintained.

At the top of the bedrock formations, the temperature distribution in the EuGF is lower than that in the previous simulations, with values ranging from 25 to 50 °C (Fig. 8c). Along the fractures, the temperature is unchanged, with a maximum of 25 °C. Consequently, the maximum values simulated at 200 m depth are lower than the mean value measured by thermal logs (Table 6 and Fig. 10c), while the temperature simulated at greater depths is not within the range of the available experimental measurements. This is also highlighted by the temperature map at a depth of 1,000 m, in which the thermal anomaly is partially developed (Fig. 9c).

The flow rate through the Cauchy-type BC ranges from 0.4 to 0.7 M m^3^/y (Fig. 11c). Here, the implemented structural setting is unable to produce a groundwater outflow comparable with the historical discharge of the thermal springs (2.6 M m^3^/year).

#### Simulation neglecting the influence of all tectonic features

In the last simulation, to evaluate the role played by the structural settings on the development of the EuGS, all DEs representing the faults and fractures were removed. Consequently, only the EPM approach was considered.

At the top of the domain (Fig. 8d), the maximum temperature is below 50 °C, while at 1,000 m depth (Fig. 9d), the increase in temperature is irrelevant (54.7 °C; Table 6). The maximum temperature in the aquifer near the EuGF is lower than 55 °C. Thus, the measurements taken in the exploitation wells do not validate the simulation results. In fact, the simulated Tmax can be considered within the range of variations in the experimental temperature at the top of the bedrock formation (200 m); at greater depths, the simulated Tmax does not match the thermal log temperature but has a discrepancy that reach 30 °C (Table 6 and Fig. 10d). Consequently, a general temperature reduction with respect to the RS is evident.

In addition, the outflow from the Cauchy-type BC is the lowest among all simulations tested. It ranges from 0.1 to 0.2 M m^3^/year (Fig. 11d) and does not match the spring flow rate in the EuGF area.

## Discussion

The renewability of a geothermal system is mainly related to the geological and hydrogeological processes that permit its development. An accurate numerical model that incorporates the main features affecting a geothermal system can be a profitable tool for evaluating these processes. The hydrogeological reconstruction of the EuGS in a numerical environment was accomplished by a 3D unstructured mesh. Hydrostratigraphic units and regional and local faults and fractures were reproduced by the explicit discrete fracture approach. The numerical RS results establish that the simulated temperature in the thermal reservoir corresponding to the EuGF is comparable to the temperature inferred by thermal logs. In particular, the simulated Tmax is within the range of values obtained by experimental measurements. In addition, the groundwater outflow simulated in the interaction zone shows values alike the flow rate of the Euganean thermal springs. These results indicate that the implemented geological framework and the assigned hydraulic and thermal properties, in addition to the applied boundary conditions, validate the existence and development of the Euganean geothermal resource.

To assess the renewability of the EuGS, several additional simulations were performed that tested the fluid and heat recharge of the system (as external forces of the geothermal system) and the role of structural features (intrinsic features of the system). The simulations of the first group confirm that the recharge area cannot be located west of the SV fault in the Piccole Dolomiti Mountains (Piccoli et al., 1973) because such a configuration hinders the southward migration of infiltrated water. The simulation with a normal crustal heat flow (60 mW/m^2^) shows a slight temperature reduction (with a maximum of 15 °C for Tmax) compared to the RS. Based on these results, we can conclude that the recharge area located east of the SV fault (Sette Comuni and Tonezza Plateaus) and the presence of an anomalous crustal heat flow at the base of the domain, are two important factors contributing to the development of groundwater flow and water temperature in the EuGS and are also controlling its renewability. In the second group of simulations, the DEs representing regional- and local-scale faults and fractures were progressively removed. The results portray a decrease in the simulated values to a maximum of 45 °C for Tmax (Tables 5 and 6). In addition, the modeled outflow is only 3–4% of the outflow in the RS since it is guaranteed only by the bedrock. Neglecting only the NNW-trending faults (SVFS) while maintaining the regional faults (R1, R2, R3, and R4 in Fig. 1) and the “hill-type” fractures in the relay ramp (RRFs in Fig. 3b), the modeled Tmax decreases by approximately 12 °C with respect to the Tmax in the RS (Tables 5 and 6). Although the temperature distribution in this scenario can be considered acceptable, the outflow from the interaction zone is 13.5–20.9% of the total outflow simulated by the RS. This aspect confirms the importance of the SVFS in the groundwater flow from the recharge area toward the interaction zone, with the SV fault acting as the fundamental structural element in controlling the renewability of the EuGS. Similarly, in the scenario maintaining only the local fracture mesh (RRFs in Fig. 3b) and without all of the regional faults (SVFS, R1, R2, R3, and R4 in Fig. 1), the flow rate through the interaction zone is 10.8–16.3% of the outflow in the RS. Moreover, Tmax is 29.9–40.2 °C lower than the maximum temperature in the RS (Tables 5 and 6).

In terms of temperature, the main elements that drive the development of a thermal anomaly similar to that observed in the field are relay ramp faults and fractures. Faults and fractures in the interaction zone favor the onset of convective transport of thermal energy from the bottom to the top of the reservoir with progressive temperature homogenization in the aquifer. As shown by the vertical profiles (Figs. 7 and 10), this convective motion is able to produce high water temperatures (86 °C) at shallow depths. This ability explains the similar temperatures reached by exploitation wells despite their different depths. As shown by the vertical profiles (Fig. 7), the temperature increment is not preferentially related to the depth at which the temperature is measured. In fact, Tmax ranges from 82.1 to 96.5 °C in an 800-m depth interval (200–1000 m; Table 5). The low thermal gradient is related to the existence of convective forces leading to an adiabatic thermal profile in the aquifer. This convective motion is structurally linked to the presence of relay ramp faults and fractures, which locally increase the permeability of the bedrock formations and are also favored by slightly anomalous regional crustal heat flow beneath the area. Inspection of the maps in Fig. 6 suggests that the temperature distribution is mainly controlled by the regional fault R3 and the “hill-type” fractures deforming the relay ramp. Consequently, the EuGS can be defined as a “convective-dominated non-magmatic geothermal play” according to the classification of geothermal systems proposed by Moeck (2014).

Considering the outflow simulated by the RS and subsequent additional simulations, it is possible to determine the contribution of each feature of the EuGS on the groundwater flow. This evaluation assumes that the flow rate from the interaction zone simulated by the RS represents 100% of the total amount of outflow. Therefore, by comparing the results of the additional simulation with those derived from the RS, it is possible to quantify the influence of each feature on the overall groundwater budget. The difference in the groundwater outflows from the interaction zone can be related to the structural elements that were neglected during each additional simulation. For example, the simulation without the SVFS enables quantification of the contribution from the SVFS, while the simulation without the regional and local faults and fractures allows evaluation of the role of these preferential flow paths on the groundwater flow. In particular, the importance of the local fracture mesh is corroborated by the results of the simulation in which all regional faults (i.e., SVFS and relay ramp faults) were removed.

Groundwater flow is mainly controlled by the SVFS acting as a preferential flow path between the recharge and discharge areas. The SVFS guarantees 79% of the total outflow through the interaction zone. The contributions of the faults in the relay ramp (R1, R2, R3, and R4) and the fractures (RRFs) are 4.7% and 11.6%, respectively. Finally, the role played by the bedrock formations in the total outflow is only 4.7%.

This work has furthered the modeling results presented by Pola et al. (2020). The main difference between the two models is the geological framework adopted as a base for the numerical simulations. In Pola et al. (2020), the hydrogeological setting was schematized with a classical “block-like” mesh in which the hydrogeological features and the structural elements were subjected to geometric simplifications. In addition, the recharge area and, consequently, the real extension of the hydrogeological system were not considered. Furthermore, the distribution of parameters evaluated by Pola et al. (2020) was used as a starting point for the RS, which was subsequently subjected to calibration to assess the temperature distribution in the EuGF and the flow rate through the interaction zone.

The numerical model presented in this paper considers the hydrogeological settings affecting the EuGS by a detailed 3D hydrogeological reconstruction with an unstructured mesh. Because of this, the main hydrogeological features (in terms of variations in the thicknesses and dips of the units and their structural deformations) and tectonic settings (in terms of the strikes and dips of the regional- and local-scale faults and the related damage zone) were reproduced. Given that the model was developed by adopting a reliable hydrogeological-based model domain, the results obtained in this study provide a more detailed understanding of the hydrogeological processes underlying EuGS renewability.

From a numerical point of view, comparing the results of the additional simulations that tested the structural controls underlines the effectiveness of the DEs in reproducing the analyzed geothermal system. Numerically, this process confirms that an explicit discrete fracture approach (EPM and DEs) is more suitable to simulating complex and heterogeneous systems such as those composed of fractured rocks (Garzonio et al., 2014; Pola et al., 2020; Renz et al., 2009).

## Conclusions

Since geological processes are primary controls on the development of geothermal systems, renewability of geothermal systems needs to be evaluated by considering the geological features characterizing the system. Numerical modeling is a beneficial instrument for reproducing and evaluating processes in a natural system. Due to geological controls on renewability, a reliable numerical model should include a solid representation of the geological framework as a base for numerical simulations. Detailed geological/hydrogeological models can be accurately reproduced by unstructured grids, avoiding the geometrical constraints of a rigid structured grid that is suitable for very simple conceptual hydrogeological models. A more reliable reproduction of the real hydrogeological setting is fundamental in a fault-controlled geothermal system, as is true for the case study presented in this paper.

The results achieved by the numerical model simulations give the following conclusion: (i) the tectonic structures, in particular the high-angle faults of the SVFS, are fundamental connections between the recharge area and the exploitation field (EuGF); (ii) the simulation without the SVFS permits evaluation of the contribution given by the SVFS, while the simulation in which all the tectonic elements were removed allows evaluation of the role of the local dense fracture in deforming the bedrock in the EuGF and its surroundings; (iii) the anomalous regional crustal heat flow ranging from 70 to 100 mW/m^2^ is essential for simulating the experimental temperature distribution in the EuGF; and (iv) all the previously stated factors along with an adequate recharge of the system permit assessment of the EuGS renewability.

The regional EuGS numerical model, which is essentially finalized to reproduce a reliable conceptual hydrogeological model, can be used as a starting point for assessing possible exploitation scenarios of the Euganean thermal resource. Maintaining the current utilization of the Euganean thermal resource for recreational purposes, heating buildings and eventually for electricity purposes could be considered a future goal. This strategy would contribute to substituting current conventional fossil fuels for a natural renewable resource, where the combination of geological factors provides sustainability for geothermal energy production.
